# The Panel Fee Inquiry

**Published:** 1924-01

**Authors:** 


### THE PANEL FEE ENQUIRY.

THE decision which was arrived at by the doctors' representatives at their special Panel Conference, to accept the finding of a Court of Inquiry rather than the Minister's offer of a fee of 8s. 6d. for five years, was the result of a majority vote, 123 members of the Conference voting for the inquiry and 49 for the 8s. 6d. On the ground that this unfortunate controversy would have been got out of the way for so long a period if the five-year settlement had been accepted, we think it a matter for regret that the 8s. 6d. offer was not taken. If the terms of reference for the Court of Inquiry are examined, it will be found that the Court are not asked to report as to the appropriate fee for any particular period. Indeed, we do not see how, with the changing economic conditions, any amount could well be fixed scientifically or judicially for a longer period than one year. It is clear from a perusal of the report of the discussion at the Panel Conference that doctors are not so keen on a long-period settlement when it is for an amount less than they think that they ought to have. It is also not to be gainsaid that there was much force in the contention of the Chairman of the Conference, Dr. Dain, that the doctors would have given themselves away rather badly if, after making out a closely-reasoned case for something like 10s. 6d. they had accepted voluntarily the fee of 8s. 6d., which they considered to be unjust, instead of having the courage of their convictions and taking their arguments before an independent Court.

### Will There be Mud Slinging ?

Dr. Brackenbury, in the course of his remarks at the Conference, anticipated that there would be mud-slinging at the inquiry. We take leave to doubt it. In the judicial atmosphere of the proceedings before the Chairman of the General Council of the Bar and the eminent banker and economist who are his colleagues in this inquiry, there will be little scope and no encouragement for the sort of irresponsible and sometimes disingenuous talk of which we have had so much in these past weeks from some of those who would have you know that they are especially jealous for the rights of the insured. We hope, and we believe, that if the Approved Societies present their point of view to the Court, they will do so temperately and will direct their observations solely to the question before it. That question is the amount which will afford adequate remuneration for the services to be rendered by general practitioners under the conditions set out in the regulations. It will be observed that it is not the service to be rendered by a particular type of medical practitioner which is in question, but the service which would be rendered by general practitioners as a class.

### A Laboured Production.

The delay which ensued in the production of the terms of reference after the announcement of the appointment of the *personnel* of the Court of Inquiry was such as to suggest that there had been much travail in the matter. The terms, in fact, follow very closely the reference to the arbitrators in 1920, but there is one significant addition—the Court are to have "due regard to the service in fact rendered under the Regulations hitherto in force." Herein, we suspect, lay the travail. If the inclusion of this phrase is a concession to the clamour of the Approved Societies, it will be of interest to see how they will address themselves to this part of the question. They, or some of them, will want to show that the service which the insured are getting is poor stuff.

### The Black Sheep.

Now, the Ministry can produoe records of individual cases of black sheep quite as bad as, or worse than, anything in the way of fresh material that any society can lay before the Court. And the doctors themselves will be the first to admit that there are such black sheep; they have asked for, and are being granted powers in the new regulations to initiate complaints through the Panel Committees against defaulting doctors, the better to get rid of the black sheep. The number of cases, however, of formal complaints established where penalties have been inflicted, as we have more than once pointed out in these columns, is an insignificant number. They may be insufficient evidence of the extent to which ground for complaint exists. But are the Court going to listen to such statements as: "We hear all sorts of complaints from our members," or "Doctors on the panel treat their patients very cavalierly," or "There is a slackness about the whole service, and this is common knowledge." The great majority of general practitioners are on the panel, and such statements are an indictment of the whole medical profession, just in the same way as statements that lawyers are notorious rogues or that the working classes are a lazy, thriftless lot. If a general indictment is not to be accepted, how many days and nights are the Court to sit and examine samples of the insured population marshalled by the societies, and how many more days and nights to listen to those assembled by the medical men to prove what good fellows the doctors are?

### A General Practitioner Service.

As to the scope of the panel doctor's obligation, there has recently been a very disingenuous attempt to separate a statement made by Dr. Brackenbury from its context, and in that way to show that doctors have no intention of treating their insured patients as well as their private patients. We, therefore, think that, in fairness, publicity should be given to a recent statement made by Dr. Brackenbury at the special Panel Conference. His Committee had offered to the Minister, he said, the fullest, freest and most effective service that could be given; a specialist service, whether rendered by a specialist or by a general practitioner who was a specialist in that particular branch, was outside the contract, but with that exception they offered the Minister the best they could. It stands to reason that specialist services cannot be given to individual insured persons until they are available generally. But as to the scope of the general practitioner service, if doctors in the mass will only do their best to "deliver the goods" in the sense indicated by Dr. Brackenbury, we shall not hear so much of the panel doctor as a separate entity.

